# Prevalence of Extended-Spectrum Beta-Lactamase Producer Gram-Negative Rods and Associated Factors Among Patients With Wound Infection at University of Gondar Comprehensive Specialized Hospital, Northwest Ethiopia

**DOI:** 10.1155/2024/1478975

**Published:** 2024-11-11

**Authors:** Sara Tesfaye, Wudu Tafere, Wondwossen Abebe, Yitayih Wondimeneh

**Affiliations:** ^1^Department of Medical Microbiology, School of Medicine, College of Medicine and Health Sciences, Jigjiga University, Jigjiga, Ethiopia; ^2^Department of Medical Microbiology, Amhara National Regional State Public Health Institute, Bahirdar, Ethiopia; ^3^Department of Medical Microbiology, School of Biomedical and Laboratory Sciences, College of Medicine and Health Sciences, University of Gondar, Gondar, Ethiopia

**Keywords:** ESBL, Gram-negative rods, UOGCSH, wound infection

## Abstract

**Background:** Multidrug-resistant (MDR) bacteria have significantly affected the management and treatment of wound infections globally. Data on the prevalence of MDR bacterial profiles that cause wound infections in Ethiopia are scarce. Therefore, this study aimed to determine MDR as well as extended-spectrum beta-lactamase production profiles of Gram-negative rods that are difficult to treat with conventional antibiotics and that cause wound infections.

**Objective:** The aim of the study was to determine the prevalence of extended-spectrum beta-lactamase producer Gram-negative rods and associated factors among patients with wound infection at University of Gondar Comprehensive Specialized Hospital, northwest Ethiopia.

**Materials and Methods:** This hospital-based cross-sectional study was conducted at University of Gondar Comprehensive Specialized Hospital between May and July 2022. Convenience sampling was used to recruit 228 participants. Swabs from different wound types were inoculated onto the MacConkey agar and blood agar plates and incubated overnight at 37°C for 24 h. Biochemical tests were performed on isolated colonies for the identification of bacterial species based on their biochemical reaction. Antimicrobial susceptibility tests were performed using the disk diffusion technique as per the standard Kirby–Bauer method by using Muller–Hinton agar, and the zone of inhibition was interpreted as resistant, intermediate, and sensitive as per the recommendation of Clinical Laboratory Standard Institute. Isolates were tested against ceftriaxone, cefotaxime, and ceftazidime for extended-spectrum beta-lactamase screening using the Kirby–Bauer disk diffusion method, and combined disk tests were applied for phenotypic confirmatory test of extended-spectrum beta-lactamase producing isolates.

**Result:** Of 228 study participants, 162 (71.1%) were culture-positive. Among culture-positive patients, 165 Gram-negative bacteria were recovered. The most common Gram-negative isolates were *Pseudomonas aeruginosa* (47; 28.5%), followed by *Escherichia coli* (43; 26.1%) and *Klebsiella pneumoniae* (24; 14.5%). The susceptibility rates of the isolate for imipenem and tobramycin were 97.0% and 73.2%, respectively, and the overall multidrug resistance rate was 80.5%. Extended-spectrum beta-lactamase producer bacteria were also isolated. Besides, all (100%) of extended-spectrum beta-lactamase producer bacteria were MDR. Living in rural areas (AOR 5.8; 95% CI [2.01–16.7]), hospital admission (AOR 3.95; 95% CI [1.13–13.83]), antibiotic use (AOR 2.83; 95% CI [1.03–7.72]), and comorbidity (AOR 0.25; 95% CI [0.07–0.59]) were significantly associated with wound infection.

**Conclusions and Recommendations:** There was a high prevalence of Gram-negative bacterial isolates in this study*. Pseudomonas aeruginosa* (28.5%) was the predominant isolate. In addition, high rates of multidrug resistance were observed. The high level of multidrug resistance in this study implies that definitive therapy should be based on culture and susceptibility analysis to promote the rational use of antibiotics and to reduce the emergence of bacterial resistance to antimicrobials.

## 1. Introduction

Wound infections are hospital-acquired infections [1] and associated with accelerated morbidity and mortality [2, 3]. These infections may result from the simplest monomicrobial pathogen or through multiple pathogens, referred to as polymicrobial [4]. Pathogens with resistance abilities make it difficult to devise proper control plans or strategies. As a result, wound infection constitutes a chief barrier to restoration and might have a negative effect on the affected person's quality of existence as well as on the recovery charge of the wound [5].

Although the causative agent of wound infection varies depending on the location of the injury, organisms commonly identified in cases of wound infection include viruses, fungi, Gram-positive bacteria, and Gram-negative bacteria [6]. Gram-positive cocci consist of *Staphylococcus aureus (S. aureus)*, *Staphylococcus epidermidis (S. epidermidis), Streptococcus faecalis (S. faecalis)*, and *Streptococcus pyogenes (S. pyogenes);* Gram-negative bacilli, ordinarily, consist of *Acinetobacter baumannii (A. baumannii)*, *Enterobacter* species, *Escherichia coli (E. coli)*, *Proteus vulgaris (P. vulgaris)*, *Proteus mirabilis (P. mirabilis), Pseudomonas aeruginosa (P. aeruginosa)*, *Klebsiella pneumoniae (K. pneumoniae), and Klebsiella oxytoca (K. oxytoca)* [7].

The control of wound infections has become difficult owing to widespread bacterial resistance to antibiotics [8]. The emergence of antimicrobial resistance (AMR) has made the choice of empirical therapy difficult and expensive [9]. Specifically, infection with multidrug-resistant (MDR) Gram-negative isolates and extended-spectrum beta-lactamase producers poses a primary challenge in the treatment of wound infections [8, 10].

Due to the nature of their outer membrane that contains lipopolysaccharides, Gram-negative bacteria are more resistant to a wide range of antibiotics [11]. Strains which produce *β*-lactamase enzyme that hydrolyzes penicillins, cephalosporins, and aztreonam make them difficult to treat [12]. Occurrence of ESBL-producing Gram-negative bacteria is widespread and includes well-known pathogens such as *E. coli*, *K. pneumoniae*, *P. aeruginosa,* and *A. baumannii* [13]. Besides, these bacteria are increasingly recognized as important contributors to infections acquired within healthcare settings and have been documented in multiple countries [14, 15].

Extended-spectrum beta-lactamases are different variants of beta-lactamase enzymes derived from the classical beta-lactamase enzymes by mutation at one or multiple points in their gene sequences and are known to mediate resistance against all beta-lactams, especially extended-spectrum beta-lactam antibiotics including the third-generation cephalosporins [16]. The ESBL-producing bacteria are increasingly becoming a major threat for patients in the hospital, long-term care facilities, and community. The increasing drug resistance among these bacteria has made therapy difficult and led to a greater use of expensive broad-spectrum antibiotics [17].

The World Health Organization has declared AMR as one of the top ten global public health threats facing humanity and recognizes it as a major public health threat in the 21^st^ century [18]. An estimated 7,00,000 people currently die of AMR infection each year; by 2050, this number could rise to approximately 10 million per year if no action is taken [19]. More than 2.8 million AMR occur, resulting in more than 35,000 deaths per year in the United States alone [20]. In Africa, it has been approximated that 4.2 million deaths occur annually due to AMR [21]. In Ethiopia, a systematic review and meta-analysis conducted to determine the prevalence of MDR bacteria showed that the overall pooled prevalence of MDR bacteria is 70.5% [22].

In addition to the alarming data on attributed deaths, antibiotic resistance has a broader impact on the economy. The loss of capital caused by AMR is estimated to be approximately $300 billion to $1 trillion by 2050 [23]. In the United States, the associated annual additional cost of infections caused by resistant organisms is estimated to be between $21 billion and $34 billion, accompanied by more than 8 million additional days in hospital [24, 25].

Therefore, it is important to understand how the resistance changes over time in a particular locality [7]. Studies have been conducted in the study area, but updated information is required because AMR is dynamic. Therefore, continuous surveillance is necessary to guide appropriate therapies for wound infections and rational use of antimicrobial agents. Therefore, a recent study is required to determine and update the current etiologic agents and antimicrobial susceptibility patterns of the isolates. Therefore, this study was designed to provide evidence-based information regarding the antimicrobial susceptibility patterns of bacterial isolates and the associated risk factors among patients with wound infections at the University of Gondar Comprehensive Specialized Hospital, northwest Ethiopia.

## 2. Materials and Methods

### 2.1. Study Area, Design, and Period

This hospital-based cross-sectional study was conducted from May 1 to July 30, 2022, at the University of Gondar Comprehensive Specialized Hospital. The hospital is situated in Gondar town which is located 750 km from Addis Ababa in northwest Ethiopia. According to the revision of the UN urbanization prospective, the projected population size of Gondar town in 2024 was estimated to be 431,113. The hospital is one of the largest tertiary-level referral and teaching institutions in the Amhara region. The hospital provides surgical, orthopedic, medical, pediatric, gynecological, obstetric, and intensive care services. It has microbiology laboratory and gives services for more than 5 million people in the surrounding zones and nearby regions [26].

### 2.2. Source Population Sample Size and Sampling Technique

The source population consisted of patients with clinical evidence of wound infections such as discharge, pain, swelling, erythema, and foul smelling at the University of Gondar Comprehensive Specialized Hospital. The minimum sample size was calculated based on the assumption of 5% expected margin of error and 95% confidence interval, taking the prevalence of 83.9% from the previous study that was conducted in Gondar town [27] using a single population proportion formula and adding a 10% non-response rate, and the final sample size was 228 study participants. The study participants were recruited using convenience sampling.

### 2.3. Data Collection and Analysis

Training was provided to the data collector for data collection and interview techniques for one day. Data related to sociodemographic characteristics and possible risk factors of the participants were collected through face-to-face interviews using semistructured questionnaires.

### 2.4. Sample Collection, Transportation, and Processing

Pus samples from the inpatient and outpatient departments were collected using sterile cotton swabs from a variety of body areas, including the leg, head, hands, arms, shoulder, and abdomen. Furthermore, samples were drawn from closed wounds using sterile fine-needle syringes (FNSs). Each sample was appropriately labeled with the MRN, date, and time of collection. Open wound swabs were aseptically obtained after immediate surface exudates, and contaminants were removed with moistened sterile gauze and sterile normal saline solution. The dressings were cleaned with sterile normal saline after removing the dressing. Specimens were collected with sterile cotton swabs by rotating them under sufficient pressure. Subsequently, the samples were immediately placed in Amies Transport Medium (Oxoid, England), transported to the UOGCSH Microbiology Laboratory, and processed within 2 h of collection for microbiological analysis. Also, FNS samples were placed in a sterile syringe and send to the laboratory immediately. For delayed samples, Amies transport media were utilized to maintain the viability of microorganisms in the sample during transport to the laboratory for culture and analysis.

### 2.5. Isolation of Gram-Negative Bacteria

The correct isolation of bacterial isolates was achieved by following standard operating procedures for the processing of wound samples prepared by the UOGCSH Microbiology Laboratory. Upon receipt of the samples in our laboratory, smear was prepared and direct Gram stain of pus sample was performed to provide preliminary information about the types of bacteria present in the sample. Swab specimens were inoculated onto MacConkey agar (MAC) (Oxoid, England) and blood agar plates (BAPs) (Oxoid, England) and incubated overnight at 37°C for 24 h. Preliminary identification of the bacteria was performed based on the characteristics of the organisms in their colonies (size, shape, color [pigmentation], texture, elevation, and edge), and the Gram smear is prepared after the plates have been inoculated from the specimen. The results such as Gram reaction (Gram-negative), arrangements, and shape of bacteria are seen from the examinations using a microscope [28]. Biochemical tests were performed on colonies from pure cultures for the identification of the isolates. Triple sugar iron agar (Oxoid Ltd, Basingstoke [for gas production, lactose fermentation, and hydrogen sulfide production], UK), indole test (for tryptophan utilization, Simon's citrate agar [Oxoid Ltd, Basingstoke, UK] [citrate utilization test]), urease agar (Oxoid Ltd, Basingstoke, UK) (urease production test), lysine iron agar (Oxoid Ltd, Basingstoke, UK) (lysine decarboxylase test), and Motility medium (Oxoid Ltd, Basingstoke, UK) (motility test) were included in the biochemical tests for species identification [29].

### 2.6. Antimicrobial Susceptibility Test

Following bacterial identification, the antimicrobial susceptibility testing (AST) of the isolates was performed by using the modified Kirby–Bauer disk diffusion technique [30] according to the criteria set by the Clinical and Laboratory Standard Institute (CLSI) (2022). Bacterial suspension was prepared from pure colonies of a young culture by picking parts [3–5] of similar test organisms with a sterile wire loop and suspended in 0.85% sterile normal saline. The density of the suspension to be inoculated was determined by comparison with the turbidity standard of McFarland 0.5 barium sulfate solution. Then, using sterile cotton-tipped swabs, the bacterial suspension was inoculated onto Muller–Hinton agar (Oxoid, Basingstoke, Hampshire, UK) using the lawn culture method, and the inoculated plates were left at room temperature for 15 min. Then, using sterile forceps, a set of antibiotic discs were placed on the inoculated MHA.

For enterobacteriaceae, the following antibiotic disks were selected based on the CLSI guideline such as beta-lactam group (cefotaxime [CTX 30 *μ*g], ceftazidime [CAZ 30 *μ*g], and ceftriaxone [CRO 30 *μ*g]), aminoglycosides (gentamicin [GEN 10 *μ*g]) and tobramycin [TOB]), carbapenems (imipenem [IPM 10 *μ*g]), fluoroquinolones (ciprofloxacin [CIP 5 *μ*g]), phenicols (chloramphenicol [CHL 30 *μ*g]), sulfonamide (trimethoprim/sulphamethoxazole [1.25 *μ*g/23.75 *μ*g]), and piperacillin–tazobactam (100/10 *μ*g). However, for Pseudomonas aeruginosa and *A. baumannii* isolates, the following antibiotics are selected based on CLSI guidelines such as piperacillin–tazobactam, TOB, CAZ, CIP, IPM, and cefepime. The antibiotic disks used were from BD, BBL Company, USA Product. The diameters of the zones of inhibition around the discs were measured to the nearest millimeter using a clipper and classified as sensitive, intermediate, or resistant according to the CLSI recommendations [31]. MDR isolates were also identified using antibiotics from different classes. Multiple drug resistance is defined as the resistance of an isolate to at least one agent in three or more antimicrobial classes [32]. The selection of antibiotics and their dosages were determined in accordance with their accessibility to the study and the frequency with which the doctor had been prescribing them.

### 2.7. Detection Test for ESBL

Gram-negative strains were tested with CRO, CTX, and CAZ for ESBL screening using the Kirby–Bauer disk diffusion method. Isolates which showed < 22 mm for CAZ, < 25 mm for CRO, and < 27 for CTX were considered as potential ESBL strains and selected for a further phenotypic confirmatory test as described below [31]. Extended-spectrum beta-lactamase was confirmed using the combined disk test (CDT): CAZ (30 *μ*g) and CAZ (30 *μ*g) plus clavulanic acid (10 *μ*g) and CTX (30 *μ*g) and CTX (30 *μ*g) plus clavulanic acid (10 *μ*g). The zones of inhibition for the CAZ and CTX discs were compared to that of the CAZ and CTX plus clavulanic acid combination discs. An increase in the zone diameter of ≥ 5 mm in the presence of clavulanic acid was concluded as confirmed for ESBL producers [31].

### 2.8. Quality Control

All laboratory activities were performed according to the standard operating procedures. Each batch's media performance and sterility were evaluated. Five percent (5%) of the prepared culture media was randomly selected and incubated aerobically for 24 h at 37°C to minimize the sterility of the culture media. Similarly, the reference strains E*. coli* ATCC 25922 and *P. aeruginosa* (ATCC 23853) were used to evaluate the performance of the prepared culture media.

### 2.9. Data Quality Control

Data quality was assured pretested questionnaires before conducting the main study. It was used to ensure the feasibility and validity of study variables. In addition, the quality of the data was assured with a close follow-up of the completeness of the checklist on the spot by the data collectors. The principal investigator together with the supervisor supervises the data collection process. In addition, the completeness and clarity of the collected data were checked carefully and regularly by the principal investigator.

### 2.10. Data Processing and Analysis

Data were entered into Epi Info Version 7 to check data completeness and clearance and then transferred to the Statistical Package for Social Sciences (SPSS) Version 20 for analysis. Summary statistics were calculated using frequencies, means, standard deviations, and proportions for the categorical data. Pearson's chi-square test was used to observe an appropriate association between independent variables and bacterial isolates, and bivariate and multivariate logistic regression analyses were performed to determine the associated risk factors. A variable with a *p* value ≤ 0.2 in bivariate logistic regression was checked in multivariate analysis for a statistically significant association. The 95% confidence interval was determined, and risk factors in multivariate analyses were considered statistically significant at a *p* value of less than 0.05. The results are presented in tables and figures.

### 2.11. Ethical Consideration

Ethical clearance was obtained from the Ethics and Review Committee of the School of Biomedical and Laboratory Sciences, College of Medicine and Health Sciences, University of Gondar, Ethiopia. Before data collection, the study participants were informed of the study, and their written consent for participation was obtained. For child participants, assent/consent to participate was collected from their parents/guardians in a written form, and confidentiality was maintained by omitting their names and personal identifiers throughout the study.

## 3. Result

### 3.1. General Characteristics of the Study Participants

A total of 228 participants with various types of wound infections were included in this study. Of the participants, 194 (85.1%) were male. The ages of the participants ranged from 6 to 80 years, with a mean age of 28.9 ± 12.9. One hundred and seven (46.9%) of them were between 21 and 30 years old, and 156 (68.4%) of the study participants were young. The majority of the study participants (76.3%) lived in rural areas, and 124 (54.4%) of the study participants were from the outpatient department. Most of the study participants had a hospital stay of > 5 (greater than five) days (61.5%), and 155 (68%) of the participants were on antibiotics. The most commonly isolated cause of the wound was bullet injury (37.7%), followed by car accidents (24.6%), and most of the wounds were located on the leg (105; 46.1%) (Table 1).

### 3.2. Overall Culture Results and Prevalence of Bacterial Isolates

**Figure 1:**
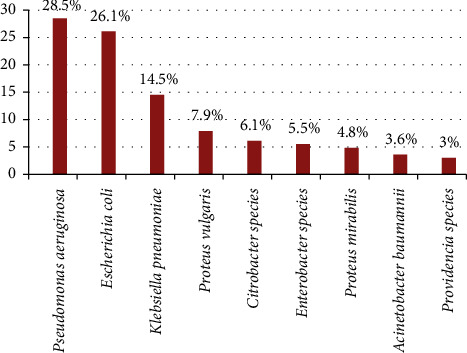
Type and frequency of Gram-negative rods isolated from wound infections from May to July 2022.

Of 228 wound samples, 162 (71.1%) were culture-positive. Three samples with two bacterial isolates yielded 165 bacterial isolates. Among the 165 bacterial isolates, *P. aeruginosa* was the most commonly isolated (47; 28.5%), followed by *E. coli* (43; 26.1%) and *K. pneumoniae* (24; 14.5%). However, *Providencia species* was the least identified Gram-negative rod, accounting for approximately five (3%) of the total isolates (Figure 1).

### 3.3. Antimicrobial Susceptibility Pattern of Bacterial Isolates

Among the 165 Gram-negative isolates, enterobacteriaceae showed highest resistance rate for ampicillin (94.5%), followed by cotrimoxazole (62.4%) and CRO (60%). However, low resistance was observed for IPM (5.4%), piperacillin–tazobactam (32.7%), and cefepime (33.3%).

Regarding to individual, *P. aeruginosa* isolates were resistant to CAZ (78.7%) and TOB (55.3%), whereas 95.7% and 61.7% of the isolates were susceptible to IPM and piperacillin–tazobactam, respectively. Similarly*, E. coli* was resistant to ampicillin (81.6%), whereas 100%, 79.1%, 78.9%, 76.3%, and 68.4% of the isolates were susceptible to IPM, piperacillin–tazobactam, GEN, cefepime, and CHL, respectively.

*Klebsiella pneumoniae* was resistant to CHL (70.8%), trimethoprim/sulfamethoxazole (75.0%), CIP (58.8%), and CRO (52.9%); however, 91.7%, 70.6%, and 62.5% of the isolates were susceptible to IPM, GEN, and cefepime, respectively. *Proteus vulgaris, Proteus mirabilis, Providencia spp, Enterobacter* spp, and *Citrobacter* showed 100% sensitivity to IPM (Table 2).

### 3.4. ESBL-Producing Isolates

**Figure 2:**
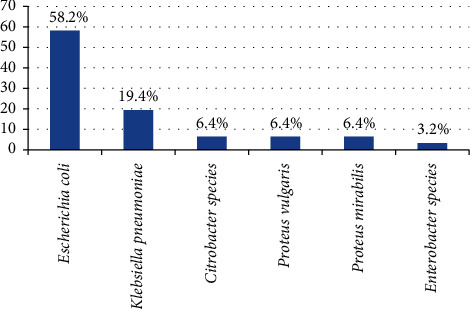
Percentage frequency of ESBL-producing Gram-negative bacterial isolates from wound infections from May to July 2022.

Among 165 Gram-negative isolates, 31 (18.8%) were ESBL producers. Among 43 isolates of *E. coli*, 18 (58.2%) were positive for ESBL production and among 24 isolates of K. *pneumoniae* 6 (19.4%) were positive for ESBL. Furthermore, *Citrobacter*, *P. vulgaris*, and *P. mirabilis* each accounted for 2 (6.4%) ESBL-producing isolates, and 1 (3.2%) *Enterobacter* isolate was also identified as producing ESBL (Figure 2).

### 3.5. MDR Pattern of Bacterial Isolates

Twelve antibiotics from eight antimicrobials were used to assess the MDR patterns of the isolates. The overall prevalence of multidrug resistance was 132 (80.5%). *Proteus vulgaris* and *Providencia* spp showed a 100% MDR rate. However, *Pseudomonas aeruginosa, K. pneumoniae, Citrobacter, Enterobacter* spp*, E.coli,* and *A. baumannii* showed 76.7%, 83.3%, 90%, 88.9%, 76.7%, and 66.7%, respectively (Table 3).

### 3.6. Characteristics of Study Participants and Their Association With Wound Infection

In bivariate logistic regression analysis, wound infection was significantly associated with residence (COR 3.96; 95% CI [1.67–9.3]), occupation (COR 2.55; 95% CI [1.14–5.73]), patient setting (COR 6.63; 95% CI [2.23–19.7]), comorbidity (COR 0.40; 95% CI [0.17–0.93]), alcohol use (COR 0.42; 95% CI [0.18–0.96]), and antibiotic usage (COR 4.44; 95% CI [2–9.9]). In a multivariate logistic regression analysis, the abovementioned factors were associated with wound infection, except for occupation. Patients who lived in rural areas were eight (AOR 5.8; 95% CI [2.01–16.7]) times more likely to develop wound infections than those who lived in urban areas. Patients who were currently admitted (inpatients) were four times (AOR 3.95; 95% CI [1.13–13.83]) more likely to develop wound infections than those diagnosed with OPD. Patients who were taking antibiotics were approximately three times (AOR 2.83; 95% CI [1.03–7.72]) more likely to develop wound infections than those who were not. Patients who were drinking alcohol were two times (AOR 2.37; 95% CI [0.39–14.28]) more likely to develop wound infection than those who were not drinking, and patients who had comorbidity were one time (AOR 1.24; 95% CI [0.53–2.93]) more likely to develop wound infection than patients who did not have comorbidities (Table 4).

## 4. Discussions

MDR Gram-negative bacteria are becoming increasingly problematic across the globe. The present study reveals information on Gram-negative bacterial profiles and their antimicrobial susceptibility patterns in wound infections, which play a decisive role in the proper selection of antimicrobial agents and prevention of future infections. In this study, the most frequently detected bacterial isolate was *Pseudomonas aeruginosa.* This observation agrees with previous studies conducted in Oman [35], Yemen [36], Ghana [37], Jimma [38], and Bahir Dar [39]. A possible reason for the high frequency of *P. aeruginosa* is that these bacteria are commonly found in hospital environments, which might increase the wound infection rate and cross-contamination among admitted patients [40]. However, a previous study from Asela [41] and Gondar [27], Ethiopia, reported *Klebsiella* species as the most prevalent agent for wound infections, and other studies conducted in Nigeria [42], Kenya [43], and Addis Ababa [44] reported *E. coli* was the predominant isolate. A possible explanation for this finding is the difference between the study participants. In those studies, most patients had surgical wounds, and most of the surgical procedures performed were laparotomy, which resulted in spillage from the gastrointestinal tract. This may be because of the natural habitat of *E. coli* is the gastrointestinal tract.

*Pseudomonas aeruginosa* showed high resistance to CRO (78.7%) and trimethoprim/sulphamethoxazole (73.3%), which is comparable to the results of a previous study conducted in Nepal and Ghana [34, 37]. However, this was higher than that reported in a previous study conducted in Gondar [22]. This may be due to the indiscriminate use of antibiotics in the hospitals. However, bacterial pathogens were highly susceptible to IPM (95.7%), piperacillin–tazobactam (70.2%), CIP (63.8%), and TOB (55.3%). This is comparable to the results documented in previous studies from Yemen [36].

In this study, IPM, GEN, CHL, and cefepime were the most effective antibiotics against *E. coli*, which is consistent with the results of previous studies conducted in Pakistan and Nepal [34, 45]. High resistance was observed to ampicillin (81.6%), tetracycline (78.9%), and cotrimoxazole (57.9%). The resistance rate observed in our study was similar to that observed in studies conducted in Nepal and Yemen [34, 36], Wollega [46], and Bahir Dar, Ethiopia [39].

*Klebsiella pneumoniae* was also sensitive to IPM (91.7%), GEN (70.8%), CHL (70.8%), cefepime (62.5%), and CIP (54.2%), which was comparable to the results documented in previous studies from Egypt [31] and Kenya [43]. This was comparable to the results of a study conducted by Jimma and Bahir Dar [38, 39].

*Citrobacter* species*, Providencia* species*, P. vulgaris, P. mirabilis,* and *Enterobacter* species showed high sensitivity to IPM (100%). The isolates showed sensitivities of 90%, 80%, 76.9%, 75.0%, and 77.8% for cefepime, respectively. These results are comparable to those of various studies conducted in Nigeria [42], Wollega [46], Jimma [38], and Bahir Dar [39].

*A. baumannii* exhibited 83.3% resistance to CIP. However, IPM and piperacillin showed 83.3% sensitivity. This finding is comparable to the results of studies conducted in Ghana and Kenya [37, 43]. Most Gram-negative bacterial isolates showed high levels of resistance to ampicillin. This may be because these are the most commonly used antibiotics, and resistance patterns have been reported in many studies [45, 46].

The MDR rate of bacteria isolated in this study was 80.5% (95% CI: 80.3–95.6). This finding was consistent with the MDR rate reported in a previous study conducted in this area [27]. However, this was lower than that reported in a study in Jimma (82.3%) [38]. This might be because empirical treatment of the isolates and/or indiscriminate and frequent use of antibiotics by practitioners, along with the unavailability of guidelines for the use of antibiotics, play a pivotal role in the emergence and spread of resistance. The type of study population, study period, and disparity in MDR definitions are also probable reasons.

In the current study, about 31 (18.8%) (95% CI: 17.3–35.8) of Gram-negative bacteria were ESBL-positive. This finding was similar to the study conducted in Nigeria (35.3%) [39], Nepal (20.7%), and Western Nepal (22.7%) [33, 47] while this finding was lower than the study conducted in Nigeria and Ethiopia (65.0% and 53.9%), respectively [48, 49]. This slight difference may be because the prevalence of ESBL-producing isolates varies geographically. The effective antibiotic for ESBL producers was found to be IPM (carbapenems) (94.8%). Studies from Nepal also showed the highest sensitivity (93.8%) to IPM [33].

Regarding possible associated risk factors, wound infections were significantly associated with residence. Patients living in rural areas are more susceptible to wound infections. This might be because patients who live in rural areas may not have access to learning and knowledge of utilizing healthcare services and using antibiotics without prescription. In addition, most of the time, people who live in that area manage themselves by farming, so their occupation may expose them to wound infection. In this study, patients who were currently admitted (inpatients) were significantly associated with wound infections. This finding is similar to those of previous studies conducted in Addis Ababa and Debre Markos, Ethiopia [50, 51]. A possible reason for this high prevalence of wound infections in admitted patients may be the cross-contamination of resistant strains in health institutions.

Patients with different comorbidities are highly susceptible to wound infections. Previous studies conducted in India [52], Harar [53], and Debre Markos [51] also showed that comorbidity was significantly associated with wound infection. A possible explanation for this is the effect of coexisting infections on weakening the immune system of patients, which makes them more susceptible to infection. This study also indicated that wound infection among alcohol consumers was higher than that among their counterparts, which is in line with a study conducted in Tigray [54] and Harar [53]. In this study, antibiotic usage was significantly associated with wound infection compared to their counterparts, which is in line with a study conducted in Debre Markos [51]. A possible explanation for this is that the treatment of this infection was empirically based on the knowledge of the etiologic agent and its susceptibility pattern. Therefore, resistant strains remain in competition, making them difficult to treat. However, in this study, age and sex were not risk factors for wound infection; however, they showed a significant association in studies conducted in Nigeria and Addis Ababa [30, 50].

## 5. Conclusions and Recommendations

Infection of wounds is a major public health concern, particularly in developing nations. Severe wound infections can result in significant losses, such as increased rates of morbidity and death. This study concluded that there was a high prevalence of culture-positive samples for Gram-negative bacterial isolates (71.1%), as well as high prevalence of ESBL (18.8%)-producing and MDR (80.5%) isolates in infected wounds. *Pseudomonas aeruginosa* was the predominant isolate. The overall high rates of isolates that produced MDR and ESBL indicated that, if necessary steps are not taken to stop their spread, their prevalence may rise in the future. Therefore, it is essential to screen for ESBL and MDR bacteria and to periodically check their patterns of antibiotic susceptibility in order to prevent and control wound infections. In addition, the bacterial isolation rate was higher in the inpatient department than in the outpatient department. Therefore, special attention should be given to infection prevention and control strategies in the hospital. Although Ethiopia is still in the early stages of AMR research, it is critical to set up surveillance programs to lower the incidence of wound infections.

## Figures and Tables

**Table 1:** Sociodemographic and clinical characteristics of study participants with wound infection among patients attending the University of Gondar Comprehensive Specialized Hospital, northwest Ethiopia, May–July 2022.

Characteristics		Frequency	Percent
Gender	Male	194	85.1
	Female	34	14.9

Age (years)	< 20	49	21.5
	21–30	107	46.9
	31–45	46	20.2
	46–60	17	7.5
	> 60	9	3.9

Educational status	Illiterate	72	31.6
	Literate	156	68.4

Occupation	Civil servant	55	24.1
	Student	35	15.4
	Farmer	89	39.1
	Private work	40	17.5
	House wife	9	3.9

Residence	Rural	174	76.3
	Urban	54	23.7

Cause of the wound	Burn	3	1.3
	Car/motor accident	56	24.6
	Ulcer	26	7.0
	Surgery	39	17.1
	Bullet	86	37.7
	Sharp material	18	7.9
	Bomb explosion	4	1.8
	Animal bite	6	2.6

Location of wounds	Leg	105	46.1
	Hand	30	13.2
	Arm	24	10.5
	Shoulder	6	2.6
	Head	7	3.1
	Abdomen	56	24.6

Patient setting	IPD	104	45.6
	OPD	124	54.4

Abbreviations: IPD, inpatient department; OPD, outpatient department.

**Table 2:** Antimicrobial susceptibility pattern of Gram-negative bacterial isolates from wound infections at University of Gondar Comprehensive Specialized Hospital, northwest Ethiopia, May–July 2022.

Bacterial isolates	AST	Antimicrobial susceptibility patterns (*n*, %)											
Gram-negative (*n* = 165)		AM	CIP	GEN	CHL	SXT	TOB	CRO	PTZ	CAZ	CTX	FEP	IMP
*P. aeruginosa* (*n = 47*)	S	Nt	30 (63.8)	Nt	Nt	Nt	21 (44.7)	Nt	29 (61.7)	10 (21.3)	Nt	23 (48.9)	45 (95.7)
	R	Nt	17 (36.2)	Nt	Nt	Nt	26 (55.3)	Nt	18 (38.3)	37 (78.7)	Nt	24 (51.1)	2 (4.3)

*E. coli* (*n = 43*)	S	8 (18.4)	28 (65.8)	33 (76.3)	35 (81.6)	18 (42.1)	26 (60.5)	26 (60.5)	34 (79.1)	24 (55.3)	29 (68.4)	34 (78.9)	43 (100)
	R	35 (81.6)	15 (34.2)	10 (23.7)	8 (18.4)	25 (57.9)	17 (39.5)	17 (39.5)	9 (20.9)	19 (44.7)	14 (31.6)	9 (21.1)	0 (0.0)

*K. pneumoniae* (*n = 24*)	S	Nt	13 (54.2)	17 (70.8)	17 (70.8)	9 (37.5)	11 (45.8)	11 (45.8)	13 (54.2)	9 (37.5)	12 (50.0)	15 (62.5)	22 (91.7)
	R	Nt	11 (45.8)	7 (29.2)	7 (29.2)	15 (62.5)	13 (54.2)	13 (54.2)	11 (45.8)	15 (62.5)	12 (50.0)	9 (37.5)	2 (8.3)

*Citrobacter* spp (*n = 10*)	S	Nt	6 (60.0)	10 (100)	6 (60.0)	4 (40.0)	7 (70.0)	4 (40.0)	7 (70)	7 (70.0)	4 (40.0)	9 (90.0)	10 (100)
	R	Nt	4 (40.0)	0 (0.0)	4 (40.0)	6 (60.0)	3 (30.0)	6 (60.0)	3 (30)	3 (30.0)	6 (60.0)	1 (10.0)	0 (0.0)

*P. vulgaris* (*n = 13*)	S	0 (0.0)	7 (53.8)	9 (69.2)	5 (38.5)	5 (38.5)	11 (84.6)	2 (15.4)	8 (61.5)	6 (46.2)	7 (53.8)	10 (76.9)	13 (100)
	R	13 (100)	6 (46.2)	4 (30.8)	8 (61.6)	8 (61.5)	2 (15.4)	11 (84.6)	5 (38.5)	7 (53.8)	6 (46.2)	3 (23.1)	0 (0.0)

*P. mirabilis* (*n = 8*)	S	0 (0.0)	6 (75.0)	4 (50.0)	2 (25.0)	3 (37.5)	5 (62.5)	5 (62.5)	5 (62.5)	4 (50.0)	6 (75.0)	6 (75.0)	8 (100)
	R	8 (100)	2 (20.0)	4 (50.0)	6 (75.0)	5 (62.5)	3 (37.5)	3 (37.5)	3 (37.5)	4 (50.0)	2 (25.0)	2 (25.0)	0 (0.0)

*Providencia* spp (*n = 5*)	S	0 (0.0)	3 (60.0)	5 (100)	1 (20.0)	2 (40.0)	5 (100)	1 (20.0)	2 (40.0)	3 (60.0)	3 (60.0)	4 (80.0)	5 (100)
	R	5 (100)	2 (40.0)	0 (0.0)	4 (80.0)	3 (60.0)	0 (0.0)	4 (80.0)	3 (60.0)	2 (40.0)	2 (40.0)	1 (20.0)	0 (0.0)

*Enterobacter* spp (*n = 9*)	S	1 (11.1)	7 (77.8)	4 (44.4)	5 (55.6)	6 (66.7)	8 (88.9)	5 (55.6)	8 (88.9)	5 (55.6)	5 (55.6)	7 (77.8)	9 (100)
	R	8 (88.9)	2 (22.2)	5 (55.6)	4 (44.4)	3 (33.3)	1 (11.1)	4 (44.4)	1 (11.1)	4 (44.4)	4 (44.4)	2 (22.2)	0 (0.0)

*Acinetobacter baumannii* (*n = 6*)	S	Nt	1 (16.7)	3 (50.0)	Nt	2 (33.3)	3 (50.0)	2 (33.3)	5 (83.3)	2 (33.3)	1 (16.7)	2 (33.3)	1 (83.3)
	R	Nt	5 (83.3)	3 (50.0)	Nt	4 (66.7)	3 (50.0)	4 (66.7)	1 (16.7)	4 (66.7)	5 (83.3)	4 (66.7)	5 (16.7)

Total resistance isolates		156 (94.5%)	64 (38.7%)	54 (33.9%)	71 (43.1%)	103 (62.4)	68 (41.2)	99 (60.0%)	54 (32.7%)	95 (57.5%)	78 (47.3%)	55 (33.3%)	9 (5.4%)

Abbreviations: AMP, ampicillin; AST, antimicrobial susceptibility test; CAZ, ceftazidime; CHL, chloramphenicol; CIP, ciprofloxacin; CRO, ceftriaxone; CTX, cefotaxime; FEP, cefepime; GEN, gentamicin; IMP, imipenem; Nt, not tested; PTZ, piperacillin–tazobactam; R, resistant; S, sensitive; SXT, trimethoprim/sulphamethoxazole; TOB, tobramycin.

**Table 3:** Multidrug-resistant pattern of bacterial isolates from wound infections attending University of Gondar Comprehensive Specialized Hospital, northwest Ethiopia, May–July 2022.

Class of antibiotics	*P. aeruginosa* [33]	*E. coli* [34]	*K. pneumoniae* [24]	*Citrobacter* spp [10]	*P. Vulgaris* [13]	*P. mirabilis* [8]	*Providencia* [5]	*Enterobacter* [9]	*Acinetobacter *[6]
3 (AMP, SXT, CIP)	—	3	—		1	1	—	3	—
3 (AMP, CIP, CTO)	—	4	—		2	0	1	2	—
4 (AMP, SXT, CIP, CRO)	—	7	3		2	2	2	—	—
4 (CIP, CRO, CAZ, FEP)	5	—	—	—	—	—	—	—	
3 (CIP, CAZ, TOB)	5	—	—	—	—	—	—	—	
4 (SXT, CIP, TOB, FEP)	2	1	—	—		—	—	—	—
4 (GEN, SXT, CIP, CHL)	—	—	3	4	2	—	—	2	
4 (SXT, CRO, CIP, FEP)	3	2	4	—		2	—	—	—
5 (AMP, SXT, CIP, CTX, GEN)		1	—	—			—	—	2
5 (AMP, SXT, CIP CAZ, CRO)	2	4	2		3	2	—	—	—
6 (AMP, SXT, CPR, CHL, CAZ, PTZ)		1	1	—		—	—	—	—
5 (SXT, CRO, CTX, CAZ, PTZ)	2	1	2	—	1	—	—	—	—
5 (SXT, CPR, CRO, CAZ, FEP)	3	3	2	4	1	—	—	1	—
6 (SXT, CIP, CRO, CAZ, FEP, TOB)	5	—	1	—	1	—	2	—	—
6 (CPR, CRO, CAZ, FEP, TOB, IMP)	6	2	1	1		—	—	—	2
7 (CIP, CRO, CAZ, CTX, FEP, TOB, IMP)	3	4	1	—		—	—	—	—
Total MDR isolates	36 (76.7%)	32 (74.4%)	20 (83.3%)	9(90%)	13(100%)	7(87.5%)	5(100%)	8(88.9%)	4(66.7%)

Abbreviations: AMP, ampicillin; AST, antimicrobial susceptibility test; CAZ, ceftazidime; CHL, chloramphenicol; CIP, ciprofloxacin; CRO, ceftriaxone; CTX, cefotaxime; FEP, cefepime; GEN, gentamicin; IMP, imipenem; PTZ, piperacillin–tazobactam; R, resistant; S, sensitive; SXT, trimethoprim/sulphamethoxazole; TOB, tobramycin.

**Table 4:** Bivariate and multivariable analyses for factors associated with wound infection of study participants at University of Gondar Comprehensive Specialized Hospital, northwest Ethiopia, May–July 2022.

Variables		Wound infection status		COR (95% CI)	*p* value	AOR (95% CI)	*p* value
		Yes	No				
Occupation	Yes	111	10	2.55 (1.14–5.73)	0.023	1.51 (0.58–3.8)	0.390
	No	87	20	1^∗^	1^∗^	1^∗^	1^∗^

Residence	Rural	155	19	8.81 (1.67–9.3)	0.002	5.8 (2.01–16.7)	0.042
	Urban	25	27	1^∗^	1^∗^	1^∗^	1^∗^

Patient setting	IPD	101	3	9.37 (2.23–19.7)	0.018	3.95 (1.13–13.83)	0.001
	OPD	97	27	1^∗^	1^∗^	1^∗^	1^∗^

Known comorbidity	Yes	84	3	6.63 (1.25–12.4)	0.019	1.24 (0.53–2.93)	0.003
	No	114	27	1^∗^	1^∗^	1^∗^	1^∗^

Alcohol usage	Yes	34	10	2.41 (1.8–9.56)	0.041	2.37 (0.39–14.28)	0.007
	No	164	20	1^∗^	1^∗^	1^∗^	1^∗^

Antibiotic usage	Yes	148	12	4.44 (2–9.9)	< 0.001	2.83 (1.03–7.72)	0.042
	No	50	18	1^∗^	1^∗^	1^∗^	1^∗^

*Note:*1^∗^ = reference group, comorbidity = (respiratory disorder, cardiac disorder, diabetes, hypertension, malignancy).

Abbreviations: IPD, inpatient department; OPD, outpatient department.

## Data Availability

All data generated or analyzed during this study were included in this article.

## Nomenclature

AMR: Antimicrobial resistance
ATCC: American Type Culture Collection
CLSI: Clinical and Laboratory Standard Institute
MDR: Multidrug resistance
SPSS: Statistical Package for Social Sciences
UOGCSH: University of Gondar Comprehensive Specialized Hospital.
